# Young and naïve B cells are a diagnostic pitfall in pediatric tonsillectomies

**DOI:** 10.1093/jscr/rjae622

**Published:** 2024-11-26

**Authors:** Sophie Tillotson, Ping Shi, Elizabeth Ray, Robert P Seifert

**Affiliations:** College of Medicine, University of Florida, 1600 SW Archer Rd, Gainesville, FL 32610, United States; College of Medicine, University of Florida, 1600 SW Archer Rd, Gainesville, FL 32610, United States; Penn State Health Milton S. Hershey Medical Center, 500 University Dr, Hershey, PA 17033, United States; Department of Pathology, Immunology and Laboratory Medicine, College of Medicine, University of Florida, 1600 SW Archer Rd, Gainesville, FL 32610, United States; Department of Pathology, Immunology and Laboratory Medicine, College of Medicine, University of Florida, 1600 SW Archer Rd, Gainesville, FL 32610, United States

**Keywords:** tonsillar marginal zone hyperplasia, mucosa associated lymphoid tissue lymphoma, light chain restriction, pediatric, polyclonal B cell

## Abstract

Tonsillar marginal zone hyperplasia may mimic mucosa-associated lymphoid tissue lymphoma, a rare diagnosis in children. Histologically, both entities can demonstrate expansion of the marginal zone with disruption of follicular architecture. However, marginal zone hyperplasia may appear polyclonal by flow cytometry. We present two pediatric tonsillectomy cases with tonsillar marginal zone hyperplasia and discuss the diagnostic challenges this poses in the pediatric population. Both tonsillectomies demonstrated expansion of marginal zones with partial architectural effacement, and flow cytometric analysis of both cases detected lambda light chain restricted, CD20(bright) B cells without CD38. Authors have suggested that the lambda restricted B cells in this setting represent naïve, unmutated B cells with preferential, but polyclonal, lambda expression. Our cases are in line with this thought. While robust, BIOMED-2 primer PCR can show dominant *IgK* peaks, which may be misinterpreted. This presents a diagnostic pitfall in the workup of pediatric tonsils that community pathologists must consider.

## Introduction

This article was previously presented as a poster titled ‘Young and Naïve: Naïve B cells are a Diagnostic Pitfall in Pediatric Tonsillectomies’ at the 2020 College of American Pathologists Annual Meeting on 10 October–13 October 2020.

Mucosa-associated lymphoid tissue lymphoma (MALT lymphoma), a low-grade B cell non-Hodgkin lymphoma arising from the post-germinal center marginal zone B cells, is the most common subtype of marginal zone lymphomas (MZLs) in adults [1, 2]. MALT lymphomas have a strong association with persistent chronic infection and inflammation and typically arise in MALT generated through inflammatory or autoimmune processes. [1–3]. The initiating event in lymphomagenesis is thought to be chronic immune system stimulation generating stepwise alterations in gene expression, leading to a lack of microenvironment dependency for B cell clones [3, 4]. Most of these lymphomas are clonal by flow cytometric analysis of surface kappa/lambda light chain expression as well as IgH gene rearrangement PCR [1]. MALT lymphoma in children, though rare, has been identified, necessitating inclusion of MALT lymphoma in differential diagnoses when evaluating tonsillar hyperplasia in children.

Pediatric nodal MZL is a separately recognized entity by the WHO with morphologic and clinical features unique from the adult counterpart [1]. Pediatric nodal MZL is male dominant, with a male:female ratio of over 10:1, with over 90% of patients presenting with stage I disease of a single head or neck node [4]. Morphologically, pediatric nodal MZL shows partial to total nodal effacement with attenuation of sinuses. Many follicles lack mantle zones and show progressive transformation of germinal center-like features. These features are not commonly described in adult MZL [4]. No etiology has been reproducibly described in pediatric nodal MZL [4].

In contrast, extranodal MZL or MALT lymphoma in the pediatric population is very rare, representing <1% of pediatric lymphomas [5]. Unlike pediatric nodal MZL, pediatric MALT lymphoma shows similar clinical and morphologic findings to that of adult MALT lymphoma [4]. Like adult MALT lymphoma, it has shown associations with *Helicobacter pylori*, autoimmune disease, and possible HIV infection, with the most common sites of involvement being the ocular adnexa, salivary glands, and skin [6]. Rare cases of transformation have been reported, again recapitulating the adult variant [4].

An important but limited study published by Attygalle *et al*. [7] described six cases of ‘atypical’ marginal zone hyperplasia (MZH) of mucosa-associated lymphoid tissue, including tonsil and appendix, in children. The lymphoid tissues in that series showed follicular hyperplasia with progressive transformation of germinal center-like changes. The B cells in almost all cases showed IgM expression, abnormal CD43 expression, no CD27 expression, and all were restricted for lambda surface light chain by flow cytometric analysis with a ‘high’ KI-67 proliferation index [7]. However, PCR for IGH, IGK, and IGL rearrangement was polyclonal [7]. The patients followed in this study did not require further treatment and were healthy at long term follow-up [7].

A small series evaluating pediatric nodal MZH showed lambda surface light chain predominance in the B cells with IGH gene rearrangement studies being polyclonal [4].

The morphologic distinction between MALT lymphoma, nodal MZL, and MZH in pediatric specimens is subtle and confounded by the presence of B cells showing surface light chain restriction in MZH cases. We present two pediatric patients both showing atypical MZH.

## Case series

### Case 1

The patient was a 5-year-old boy with a prior history of Burkitt lymphoma who was diagnosed and treated with 4 cycles of chemotherapy over the course of 4 months. Following completion of chemotherapy, he experienced recurrent bilateral otitis media and recurrent tonsillitis. Rapid strep testing was performed for the four episodes of tonsillitis in the 6 months preceding surgery; all results were negative. He underwent bilateral adenotonsillectomy for treatment, and tonsillar samples were sent for evaluation by pathology. The pathology report demonstrated heterogeneous lymphoid cells, 75.86% of which were B lymphocytes that demonstrated a decreased Kappa/Lambda ratio. PCR for IGH gene rearrangements was polyclonal. FISH was negative for MYC gene locus rearrangement. The lambda predominance and polyclonality of his B cells suggested MZH, rather than lymphoma, as his diagnosis.

**Figure 1:**
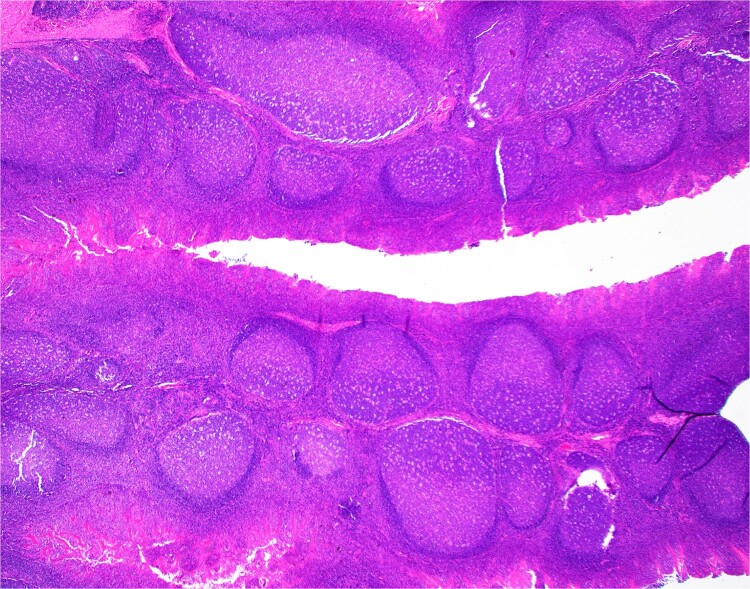
H&E section from Case 1 tonsillectomy. Representative H&E section (20×) of Case 1 tonsillectomy showing intact architecture, follicular and MZH with follicles having definitive polarity and abundant tingible body macrophages.

**Figure 2:**
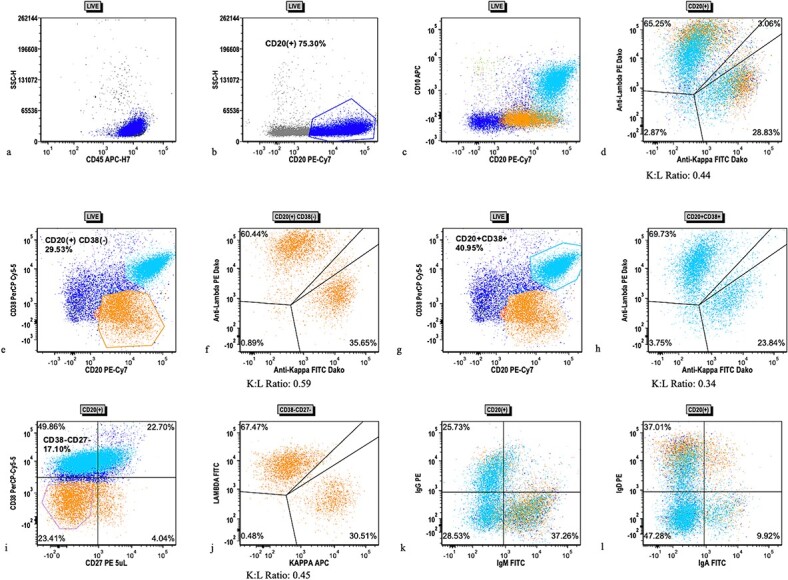
Flow cytometric analysis scatter plots from case 1 tonsillectomy. Flow cytometric analysis scatter plots showing (a) live gate with mostly CD45(+) lymphocytes, (b) CD20(+) B cells (dark blue), (c) subpopulation of CD10(+), CD38(+) B cells, (d) kappa/lambda surface light chain expression on all CD20(+) B cells, (e) gating of CD38(−) (orange) B cells, (f) kappa/lambda surface light chain expression on CD38(−) B cells, (g) gating of CD38(+) B cells (cyan), (h) kappa/lambda surface light chain expression on CD38(+) B cells, (i) CD27 expression on all CD20(+) B cells with gating of CD38(−), CD27(−) population, (j) kappa/lambda surface light chain expression on CD27(−), CD38(−) B cells, (k) IgM and IgD expression on B cells, and (l) IgG and IgA expression on B cells.

The patient’s bilateral tonsillectomy (Fig. 1) showed follicular and MZH with follicles demonstrating intact architecture, definitive polarity, and frequent tingible body macrophages. Flow cytometric analysis detected a decreased Kappa:Lambda ratio among the B cells present in both the left and right tonsil specimens, and they showed similar patterns. The Kappa:Lambda ratio ranged from 0.34 to 0.59 among differentially gated B cell populations. The Kappa:Lambda ratio remained low regardless of gating on CD10(+), CD38(+) germinal center B cells (Fig. 2h), versus CD38(−) B cells (Fig. 2f). Approximately 26.74% of all CD20(+) B cells were at least dimly CD27(+) (Fig. 2i), with the majority of B cells having a naïve immunophenotype, expressing mostly IgM (Fig. 2k), IgD (Fig. 2j), and lacking CD27. When gating on the naïve CD27(−), CD38(−) B cells, the Kappa:Lambda ratio remained low, 0.45 (Fig. 2j).

### Case 2

The patient was a 4-year-old girl with tonsillar hypertrophy and a complex infectious history of recurrent urinary tract infections and acute otitis media documented over multiple years. Urine cultures that were performed during the patient’s recurrent UTI episodes were positive for both antibiotic susceptible and resistant *Escherichia coli* and *Klebsiella pneumoniae* on different occasions. However, the patient had no clear evidence of pelvic inflammatory disease to explain her recurrent UTIs.

During hospitalization for periorbital swelling, further evaluation was pursued, investigating the patient’s recurrent infections. Labs were drawn to check vaccination response, evaluate for primary immunodeficiency and autoimmunity, and evaluate complement levels. Results indicated a normal response to vaccination. Additionally, all autoimmunity labs were within normal ranges except for SSB (La) antibody, which was one point outside of the reference range at 41 AU/ml (0–40 AU/ml). Results of her primary immunodeficiency panel were unremarkable other than revealing heterozygosity for one increased risk allele at NOD2 (p.Arg702Trp). Complement values were within normal range and, at the time of testing, all immunoglobulin levels were normal except IgG, which was slightly below reference range at 539 mg/dl (542–1358 mg/dl), and IgE, which was above reference range at 947 kU/L (≤307 kU/L). According to these results, immunodeficiency or autoimmune disease were unlikely.

In response to her recurrent infections, she underwent bilateral tonsillectomy, adenoidectomy, and bilateral tympanostomy tube placement to mediate infections and manage symptoms. Flow cytometry of tonsils demonstrated lambda predominant B cells, prompting molecular and cytogenic studies. PCR for IGH gene rearrangements was polyclonal. FISH revealed variable chromosome 14q deletion and a constitutional inversion of chromosome 9. The diagnosis was felt to be most suggestive of tonsillar MZH due to the polyclonal B cells detected upon flow cytometry.

**Figure 3:**
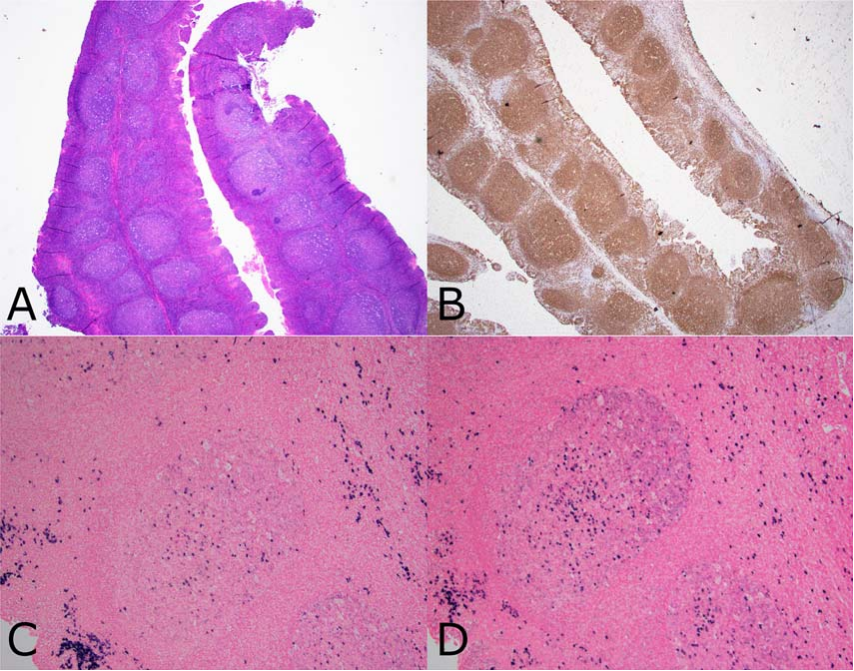
Composite photomicrograph from case 2 tonsillectomy. Composite photomicrograph from case 2 tonsillectomy showing (a) tonsil (H&E 20×) with follicular and MZH showing intact architecture and well polarized follicles. (b) CD20 immunohistochemistry (20×) showing B cell enrichment of follicles. (c and d) show the same follicle (100×) having lambda light chain predominance (d), including many dark zone B cells having diffuse lambda reactivity without as much kappa (c).

**Figure 4:**
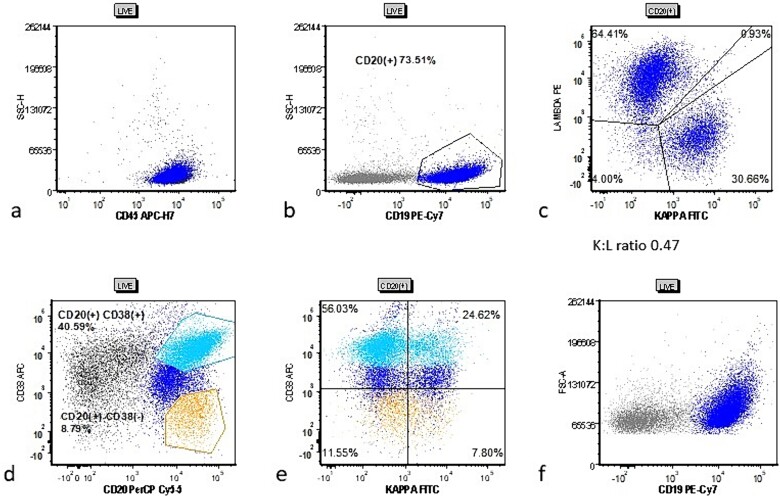
Cytometric analysis scatter plots from case 2 tonsillectomy. Case 2 flow cytometric analysis scatter plots showing (a) live gate with mostly CD45(+) lymphocytes, (b) CD20(+) B cells (dark blue), (c) B cells showing a decreased Kappa:Lambda ratio of 0.47, (d) gating for CD38(−) (orange) and CD38(+) (cyan) B cells, and (e) the CD38(−) and CD38(+) populations show more pronounced lack of kappa surface light chain.

The patient’s bilateral tonsillectomy specimen showed follicular and MZH with intact architecture (Fig. 3). A decreased overall Kappa:Lambda ratio of 0.47 was detected by flow cytometric analysis (Fig. 4c). Both CD38 negative and CD38 bright positive B cell populations show a distinct lack of Kappa surface light chain expression (Fig. 4d and e).

## Discussion

In this report, we described two pediatric patients who underwent tonsillectomy and were found to have Lambda predominant but polyclonal B-cells by flow cytometry. Tonsillectomy was performed on both patients due to recurrent infections, though they both had interesting individual presentations. One patient had a past medical history of Burkitt lymphoma and presented with frequent tonsilitis and recurrent acute otitis media after finishing chemotherapy. The other had recurrent UTIs and acute otitis media in the absence of pelvic inflammatory disease, immunodeficiency, or any diagnosed autoimmune condition. Regardless, both patient presentations are at least suggestive of immune system abnormalities: patient 1 being post-chemo and patient 2 having recurrent unusual infections. To date, both patients are alive and healthy.

Normally, kappa and lambda light chains of B cells are distributed in an ~2:1 ratio, and abnormal ratios, like those seen in our patients described above, are associated with immunodeficiency, infections, or autoimmune diseases [8]. Similar to the study by Attygalle *et al.* [7], the cases in our report showed lambda light-chain restriction, raising the possibility of lymphoma. While the patients in our study did demonstrate lambda light chain restriction, there was no evidence of monoclonality, as seen by the negative immunoglobulin heavy chain gene rearrangement analysis. This suggested a diagnosis of MZL was unlikely for the patients in our study. Previous reports shared similar observations [7, 9, 10], but the exact mechanism remains unclear. In newborns, the Kappa:Lambda ratio persists the same as in adults until age 3–5 months, around as long as maternal IgG remains in the peripheral blood, then decreases to its lowest value (around 1.0) [11]. It has been postulated that this altered ratio is due to the immaturity of pediatric immune systems [8]. Serial monitoring of immunoglobulins may provide more insight.

BIOMED-2 multiplex PCR assays have been used as a gold standard method for the diagnosis of most B cell neoplasms, especially when combined with immunoglobulin heavy chain (IGH) and immunoglobulin kappa light chain (IGK) [12]. In our cases, we observed a negative IGH PCR and a ‘monoclonal’ IGK gene amplification. According to the study by Zhang *et al.* [12], the IGH gene PCR combined with the immunoglobulin lambda light chain (IGL) gene may be used to improve the diagnostic sensitivity and accuracy. Moreover, the concentration, storage time, and DNA integrity can also significantly affect the test results [13]. So more reliable and valuable methods are necessary for clonality detection.

Dono *et al.* [14] stated that in tonsillar subepithelial B cells, CD27 expression was usually found on somatically mutated B cells whereas those without CD27 expression corresponded with unmutated B cells. In our study, a fraction of the proliferative B cells were CD27 negative. Furthermore, these cells co-expressed IgM and IgD, again suggesting naivety.

While our patients lacked evidence supportive of lymphoma, if they were indeed immunodeficient, they may be at increased risk for the development of lymphoma later in life. Future work should include in-depth immunophenotypic and genomic analysis of B cell subsets in the primary and secondary lymphoid organs of pediatric patients.

We discuss two pediatric patients presenting with recurrent infections who underwent tonsillectomy and were found to have lambda predominant but polyclonal B cells by flow cytometry. The initial lambda predominance was concerning for lymphoma in the setting of presumed immunodeficiency, as our first patient was status post-chemotherapy and the second had recurrent unusual infections. Further investigation, including flow cytometry, precluded the diagnosis of MZL. The presence of lambda predominant B cells in pediatric presentations of MZL, MALT lymphoma, and MZH warrants additional investigation and can pose a diagnostic challenge as MZH in the pediatric population can mimic MALT lymphoma. Identification of light-chain restriction by flow cytometry is not necessarily diagnostic of lymphoma. Carefully interpreting the histology, immunophenotype, and cytogenetic studies of B cells is highly recommended before making a diagnosis of MALT lymphoma in a pediatric patient.
